# It Pays to Go Off-Track: Practicing with Error-Augmenting Haptic Feedback Facilitates Learning of a Curve-Tracing Task

**DOI:** 10.3389/fpsyg.2016.02010

**Published:** 2016-12-26

**Authors:** Camille K. Williams, Luc Tremblay, Heather Carnahan

**Affiliations:** ^1^Rehabilitation Sciences Institute, University of Toronto, TorontoON, Canada; ^2^Faculty of Kinesiology and Physical Education, University of Toronto, TorontoON, Canada; ^3^School of Human Kinetics and Recreation, Memorial University of Newfoundland, St. John’sNL, Canada

**Keywords:** motor learning, haptic training, error-based learning, guidance, haptic assistance, error amplification, augmented feedback, path-following

## Abstract

Researchers in the domain of haptic training are now entering the long-standing debate regarding whether or not it is best to learn a skill by experiencing errors. Haptic training paradigms provide fertile ground for exploring how various theories about feedback, errors and physical guidance intersect during motor learning. Our objective was to determine how error minimizing, error augmenting and no haptic feedback while learning a self-paced curve-tracing task impact performance on delayed (1 day) retention and transfer tests, which indicate learning. We assessed performance using movement time and tracing error to calculate a measure of overall performance – the speed accuracy cost function. Our results showed that despite exhibiting the worst performance during skill acquisition, the error augmentation group had significantly better accuracy (but not overall performance) than the error minimization group on delayed retention and transfer tests. The control group’s performance fell between that of the two experimental groups but was not significantly different from either on the delayed retention test. We propose that the nature of the task (requiring online feedback to guide performance) coupled with the error augmentation group’s frequent off-target experience and rich experience of error-correction promoted information processing related to error-detection and error-correction that are essential for motor learning.

## Introduction

It is well known that the nature and scheduling of feedback is extremely important in the process of motor learning. Recent technological developments, particularly in haptics and robotics, have allowed researchers to embed novel and complex feedback presentations into training programs. Robotic training and haptics-enhanced performance are being explored in fields, such as neurorehabilitation (Kahn et al., 2006; Huang et al., 2009), surgical training (Prasad et al., 2002; Delorme et al., 2012), handwriting instruction (Vishnoi et al., 2009; Kim et al., 2013; Xiong et al., 2013), and sports training (Huang et al., 2011). The most common form of robotic or haptic training is haptic guidance; however, the term is used to refer to a variety of training strategies (Williams and Carnahan, 2014), including that which delivers forces, or assistance, on the basis of movement-induced feedback about performance (Bluteau et al., 2008; Marchal-Crespo and Reinkensmeyer, 2008; Lee and Choi, 2010; Marchal-Crespo et al., 2010). Haptic assistance most often serves to minimize errors in performance, either directly, by physically correcting or limiting movement errors, and/or more indirectly, by reducing task difficulty or highlighting the correct movement.

Error avoidance during skill acquisition seems intuitive because this is the ultimate goal of practice – acquiring the ability to perform a task with minimal errors (Johnson, 2004) and in fact, there are a couple lines of evidence that support the avoidance of errors during the learning process. The first is based on the idea that erroneous responses made during practice may be remembered and later repeated (Holding, 1970). Consequently, correct learning of the task of interest would require, not only the acquisition of new information and skills, but also the unlearning of incorrect ones (Kay, 1951). However, Holding (1970) found that despite some repetition of errors, there was little correlation between the errors made in early and later practice. Nonetheless, proponents of errorless learning believe that the experience of errors, especially early in learning, can lead to frustration, practice of undesirable behaviors which must later be unlearned, and lack of positive reinforcement (Singer, 1977), none of which are particularly beneficial for learning. Although this idea first emerged with animal studies in the context of discrimination learning (Terrace, 1963), it has since been implemented using prompts and cues (Singer and Gaines, 1975), physical guidance (Holding and Macrae, 1964, 1966; Armstrong, 1970) and task constraints (Maxwell et al., 2001; Capio et al., 2013) for learning a variety of motor skills. Studies have shown that learners who experienced minimized errors, through some form of guidance or prompting during practice, performed better during acquisition than groups who experienced trial-and-error learning conditions (Armstrong, 1970; Singer and Gaines, 1975; Singer and Pease, 1976; Wulf et al., 1998). While these learners typically experienced decrements in performance on retention and transfer tests (Armstrong, 1970; Singer and Gaines, 1975; Singer and Pease, 1976), a few studies have shown that some benefits of guided practice persist during unguided performance (Wulf et al., 1998; Marchal-Crespo and Reinkensmeyer, 2008; Marchal-Crespo et al., 2010). Furthermore, other studies that minimized errors through the low-to-high progression of task difficulty over the course of practice, have shown this errorless practice approach led to better retention and transfer (Maxwell et al., 2001; Poolton et al., 2005) as well as protected against performance decrements under secondary task loading in both adults (Maxwell et al., 2001; Poolton et al., 2005) and children (Capio et al., 2013). These benefits have been attributed to the release of working memory when learning under errorless conditions and the continued release of working memory during subsequent performance (Poolton et al., 2005).

In spite of this evidence supporting error minimization during training, there are theories that suggest we should be cautious about this approach, and in fact, support the creation of training scenarios that facilitate the commission of errors in order to enhance learning. The theory of desirable difficulties (Bjork, 1994; Linn and Bjork, 2006) posits that introducing difficulties for the learner by, for example, varying conditions of practice, utilizing distributed practice, and reducing feedback, often lead to poorer performance during training but enhanced post-training retention and transfer performance. Effortful training conditions may lead learners to be more active and engaged in training, thereby enhancing information processing and better protecting against the forgetting of new information and skills (Schmidt and Bjork, 1992; Lee et al., 1994). Furthermore, as suggested by the schema theory, the detection and correction of movement errors drive motor adaptation and motor skill acquisition (Schmidt and White, 1972). Likewise, error detection and correction processes update internal models that map movements of the limb to consequences in the environment (Lisberger, 1988; Kawato, 1990; Thoroughman and Shadmehr, 2000; Wolpert and Ghahramani, 2000). It is, therefore, logical to propose that artificial augmentation of errors may produce similar or added benefits to learning. These benefits may include: (i) increasing motivation by highlighting consequences of error; (ii) increasing the detection and correction of small errors; and (iii) boosting the signal-to-noise ratio for sensory feedback (Wei et al., 2005). A few studies have shown that error-augmentation or noise-like haptic disturbance may be just as beneficial as haptic assistance for learning path-following (Chen and Agrawal, 2013), pursuit tracking (Powell and O’Malley, 2012; Lee and Choi, 2014), and target-hitting tasks (Powell and O’Malley, 2012). It has also been shown that, for a golf putting task, while error augmentation had no effect on post-training performance, it did negatively impact motivation, both during and after training (Duarte and Reinkensmeyer, 2015). This is in contrast to one of Wei et al. (2005) hypothesized benefits of artificial error augmentation. Nonetheless, Milot et al. (2010) demonstrated that error-amplification for timing in a pinball-like game is beneficial for initially skilled, young adult learners but not for older adults (Bouchard et al., 2015). In addition, if we look outside the realm of upper-extremity movements, studies have also shown some benefits of error augmentation in comparison to no robot assistance for a simple locomotor task, particularly for initially less skilled learners (Marchal-Crespo et al., 2014b). However, for a more complex locomotor task, it was found that error augmentation reduced errors from baseline immediately after training for initially *more* skilled learners while random noise-like perturbation reduced errors for both skilled and unskilled participants (Marchal-Crespo et al., 2014a).

Haptic feedback is particularly suited to providing information about or altering performance while movement is ongoing (i.e., concurrently). Consequently, we are particularly interested in understanding the relative benefits of these two forms of haptic feedback (assistance and error-augmentation) for continuous, trajectory-based tasks that require ongoing use of feedback, throughout movement execution, as opposed to recall of spatial trajectories which have also been studied (Feygin et al., 2002; Liu et al., 2005, 2006; Yang et al., 2008). Such tasks are relevant to numerous functional skills including steering – in the contexts of human–computer interaction (Accot and Zhai, 1999) as well as vehicle control (Chen et al., 2011; de Groot et al., 2011), drawing (Garcia-Hernandez and Parra-Vega, 2009; Kyung et al., 2009), some cases of calligraphy/handwriting (Kim and Yang, 2007; Bluteau et al., 2008; Yang et al., 2008) and surgery (Lathan et al., 2000). Steering or tracing tasks are well-studied in the field of human–computer interaction but such studies typically involve modeling performance under various conditions or evaluating performance with different input devices without concern for any practice or learning effects that may occur. While some studies have explored haptic training for these types of feedback-dependent trajectory-based tasks, only a fraction of them (Lee and Choi, 2010, 2014; Powell and O’Malley, 2012; Chen and Agrawal, 2013) have compared the learning effects of both error minimizing and error augmenting haptic feedback. However, these trajectory-based tasks are often externally-paced in the laboratory, i.e., presented with a target or fixed speed for performance, presumably to tease apart the effects of haptic training on the spatial and dynamic features of the task. However, many functional tasks performed outside the laboratory are self-paced and only one study, to our knowledge, has compared haptic error minimization and error augmentation for learning a continuous, trajectory-based, self-paced task (Chen and Agrawal, 2013). The results from this study suggested that both haptic feedback paradigms enhanced performance but there was no significant difference between them. Looking to the broader motor learning literature, it has been suggested that closed, self-paced skills are targeted to automation and should therefore benefit from guided practice, i.e., error minimization (Singer, 1977). However, the importance of online feedback and corrections for continuous skills suggest the primacy of an error-based learning mechanism which should benefit from error augmentation. In the present study, we compared practice conditions with error-minimizing haptic feedback, error-augmenting haptic feedback and no haptic feedback for learning a tracing task. We hypothesized that: (i) in comparison to error-minimizing haptic feedback, error-augmenting haptic feedback would be more beneficial for learning; and (ii) in comparison to no haptic feedback, error-minimizing haptic feedback would be detrimental for learning. We utilized a transfer design (Salmoni et al., 1984), whereby, following skill acquisition under their assigned haptic feedback condition, all participants were tested under conditions without any augmented feedback, in order to distinguish between the transient performance effects caused by our practice conditions and more permanent changes in ability, which is indicative of motor learning (Schmidt and Bjork, 1992).

## Materials and Methods

The University of Toronto’s Health Sciences Research Ethics Board approved the protocol and we recruited 27 adults with normal or corrected-to-normal vision: 24 women, 3 men, aged 22–55 years (*M* = 28.5, *SD* = 8.7), 24 of whom self-identified as right-handed while three self-identified as left-handed. All participants gave voluntary informed consent in accordance with the guidelines set out by the 1964 Declaration of Helsinki and received gift cards valued at $15 as compensation for their time.

### Apparatus

The experiment was conducted using a SensAble Phantom Omni (currently Geomagic Touch; Rockhill, SC, USA), a standard computer monitor (Dell UltraSharp^TM^ 2209WA) and a custom software program. The Phantom Omni is a three-degree of freedom, desktop haptic interface that can exert precise forces to the user through its end effector via a handheld stylus as well as measure the position of said end effector in space (Massie and Salisbury, 1994). The device allows users to feel and interact with virtual objects. Its maximum exertable force is 3.3 N and its position resolution is approximately 0.055 mm. The visual gain between displacements on the visual display and the haptic device was 1.48. The haptic device was programmed to operate in one of three feedback modes: none, spring assistance, and spring disturbance. The spring assistance mode produced a linearly increasing spring-like force that pulled the user back toward the target curve if the cursor deviated beyond a specified limit (bandwidth), while the spring disturbance mode produced a linearly increasing spring-like force that pushed the user farther from the target curve, augmenting errors, if performance deviated from the curve beyond a specified bandwidth (see below). For the assistance (i.e., error minimization) and disturbance (i.e., error augmentation) modes, haptic gain, magnitude, and channel bandwidth were established through pilot experimentations. The haptic gain represents the spring constant, *k* in the spring force equation, f = k × x, where *f* is the force exerted by the device, up to a maximum specified by the magnitude parameter, and *x* is the displacement between the cursor and its target position on the target curve. The programmable ranges for both gain and magnitude are [0, 1], representing values 0–0.5 N/mm for gain and 0–3 N for magnitude. The channel bandwidth parameter specifies the radius of the haptic feedback-free zone on either side of the target trajectory where no forces will be exerted on the user, regardless of the mode of operation. The gains for each of the experimental conditions were set to be equivalent in order to maintain the rate of change of feedback. During pilot studies, performing under the error augmentation condition with the same gain and magnitude as the error minimization condition made it very difficult to complete a trial. As a result, the haptic gain and magnitude for the error minimization condition were 0.3 N/mm and 1 N, respectively, while the gain of the error augmentation condition was kept the same (i.e., 0.3 N/mm) but the magnitude was reduced to 0.3 N. The target curve was 0.8 mm wide and the invisible channel bandwidth was 0.8 mm on either side of the target curve.

### Task

**FIGURE 1:**
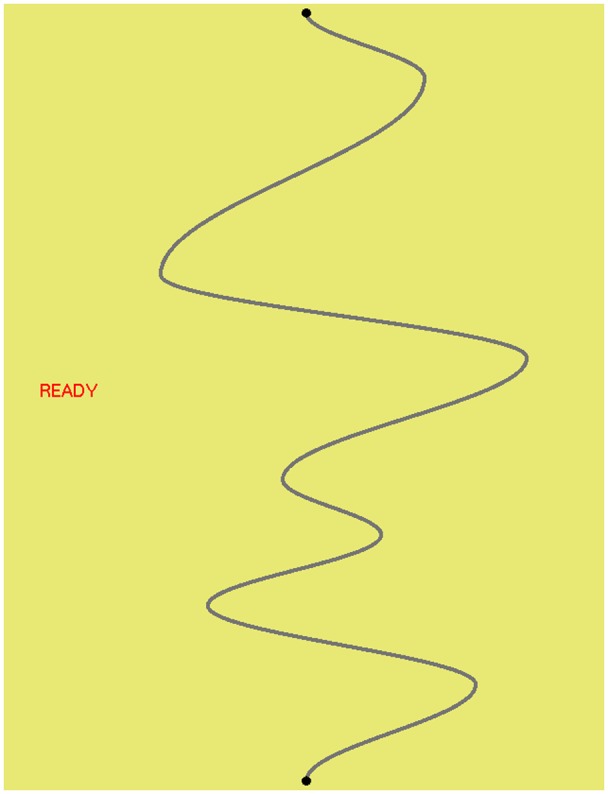
**Cropped screenshot showing the target curve used for the curve-following task**.

The position of the haptic device was adjusted for handedness and movements primarily took place in the vertical plane. Seated at a table, participants were required to grasp the device’s stylus to manipulate the corresponding cursor shown on the computer screen. The target curve comprised seven fixed control points, a start-point and an end-point, all of which were connected by sinusoidal curve segments (**Figure 1**). With respect to movement of the stylus, the amplitude of the curve was 155.6 mm from start-point to end-point, with path length 392.4 mm. With the visual gain, these measurements were 230.1 and 580.2 mm, respectively, on the computer monitor.

### Procedure

There were three practice/acquisition conditions: (i) a control condition with no manipulation of errors using the “none” feedback mode; (ii) an error minimization condition using the “spring assistance” mode; and (iii) an error augmentation condition using “spring disturbance” mode. Using the Research Randomizer website^1^, participants were randomized to one of these three conditions for practice of the task, with the sole constraint that group assignments were equal in number, (i.e., nine participants in each group). Based on this random assignment, it was assumed that all groups were equal in skill prior to training; however, this was not measured with a pre-test to avoid providing additional practice, which could be problematic for this relatively simple task. After introductory explanation of the task, participants were allowed three familiarization trials with a curve different than the one to be learned. Once participants were comfortable with the device and the task, the experimenter explained, per their group assignment, what they should expect with respect to haptic feedback when practicing the task. Participants then began the practice phase: 100 trials organized as 20 blocks (five trials/block). Once the target curve appeared on the computer screen, participants started the trial by moving the cursor to the start-point near the bottom of the screen and ended the trial by moving the cursor to the end-point near the top of the screen. The target curve and the cursor were visible for the entire trial and participants were instructed to trace the curve as quickly and as accurately as possible. After each trial, feedback regarding the tracing accuracy (a red trace of their movement superimposed over the target curve) and the movement time (a numerical value displayed in seconds to the nearest decisecond) were provided onscreen. Participants also received a numerical summary tracing error score [a numerical value, in arbitrary units (AU) where 1 AU = 0.0798 mm] after each block indicating the average tracing error for a block of trials. Ten minutes after the end of practice, participants completed an immediate retention test in which they attempted five trials of the task without any augmented haptic feedback and without summary or terminal feedback regarding tracing error or movement time. Solely the cursor and the target curve were visible throughout these retention test trials. After approximately 1 day (*M* = 1.1, *SD* = 0.4), participants returned for a delayed retention test, which was identical to the immediate retention test, and a transfer test that solely differed from the retention test by having the target curve a mirror-reversal of the one that was practiced. Prior to skill acquisition, all participants were informed that the tests following skill acquisition would not contain any augmented haptic or visual feedback about performance.

### Outcome Measures and Data Analyses

Performance was evaluated by calculating the tracing error, movement time, and samples outside the bandwidth. The tracing error (in mm) for each trial, similar to the mean modulus error (Poulton, 1974), was calculated as follows:

$$\begin{array}{c} \mathrm{Tracing}\;\mathrm{Error}=\frac{1}{N}\sum_{i=1}^{N}|e[i]| \end{array}$$

Where there are *N* samples in a trial, for each sample, the distance, *e*[*i*], between the cursor and the next untraced portion of the curve was calculated; then, all these distances were averaged to produce the tracing error for that trial. Movement time for each trial was measured as the time (in seconds to the nearest millisecond) from when the cursor was moved to the start-point, to when the cursor was moved to the end-point. We then calculated a measure of overall performance efficiency, the Speed Accuracy Cost Function: Cost Function = Tracing Error ×Movement Time (Culmer et al., 2009; Raw et al., 2012). While we were primarily interested in tracing accuracy, the cost function variable allowed us to account for the speed-accuracy trade-offs that participants would inevitably make when performing this task. As such, we could determine if better tracing accuracy was a result of increased skill or simply a slower movement. A large cost function indicated less efficient and overall, poorer, task performance.

In order to determine whether any effects of haptic feedback were simply due to participants having different amounts of feedback about their performance, we measured samples outside the bandwidth, expressed as a percentage of samples in a trial, to ascertain the amount of augmented haptic feedback that was provided on a given trial. This data was only relevant for the error minimization and error augmentation groups during the acquisition phase of the experiment. All these data (movement time, tracing error, cost function, and samples outside the bandwidth) were averaged over each block of five trials to provide 20 data points for the acquisition phase and three data points for the retention/transfer phase of the experiment.

The primary outcome measure was the cost function and we conducted a mixed model ANOVA (3 group × 20 block in acquisition) with repeated measures on the block factor for the skill acquisition data and three separate one-way ANOVAs for each of the retention and transfer tests. The cost function provides a succinct and easily understood measure of performance. However, there are multiple combinations of the component factors (movement time and tracing error) that could lead to a particular outcome. For example, an increase in cost function (i.e., a decline in performance), could be due to increased movement time, increased tracing error or both. In order to fully understand the mechanisms leading to any significant effects on the cost function, we conducted similar analyses for movement time and tracing error, as appropriate. We also conducted a mixed ANOVA (2 group × 20 block) with repeated measures on the block factor on the samples outside the bandwidth data. Because we expected that performance would improve over the course of acquisition (i.e., cost function and samples outside the bandwidth would decrease), main effects or interactions involving block in acquisition were explored using contrasts with the first block as the reference category. Main effects of practice condition or test were explored using *post hoc* comparisons using Tukey HSD as well as the Games-Howell procedure when there was concern about the homogeneity of variances, as determined by Levene’s test (Field, 2009). When Mauchly’s test indicated that the assumption of sphericity had been violated for repeated measures factors, Greenhouse-Geisser corrections were applied (all 𝜀 < 0.75) and adjusted degrees of freedom were reported. Effects for all analyses were considered statistically significant at *p* < 0.05. Effect sizes associated with *F*-tests were estimated using partial eta squared values (η_p_^2^).

## Results

Results of the analysis of the acquisition and immediate retention data demonstrated the immediate effects of our error manipulations during and shortly after practice, while the results for the delayed retention and transfer tests were used to infer learning effects from our haptic feedback manipulations during acquisition.

Due to technical difficulties, one participant in the error augmentation group completed only 15 practice blocks. Consequently, this participant’s data were excluded from the statistical analyses of acquisition data (because these were repeated measures analyses). However, since three quarters of this participant’s skill acquisition trials were available, the data were included for the analyses of retention and transfer tests.

### Amount of Haptic Feedback Experienced During Acquisition

**FIGURE 2:**
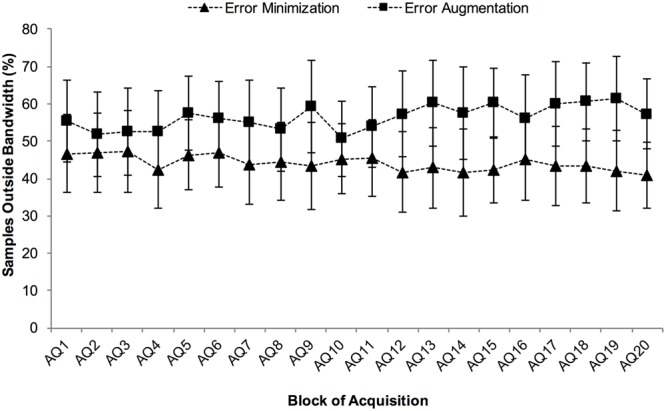
**Average percent of samples outside the bandwidth for each practice group in each block of Acquisition (AQ)**. Error bars are 95% confidence intervals.

Analysis of the proportion of samples that were outside the bandwidth (i.e., percent of the movement for which haptic feedback was received) resulted in a significant block by practice group interaction, *F*(19,285) = 2.5, *p* = 0.0001, η_p_^2^ = 0.14 (**Figure 2**). Neither the effect of practice group, *F*(1,15) = 3.6, *p* = 0.076, η_p_^2^ = 0.19, nor the effect of block, *F*(19,285) = 0.9, *p* = 0.614, η_p_^2^ = 0.06, were significant. To break down this interaction, we first performed simple contrasts with block 1 as the reference category for each practice group. This analysis did not yield any significant differences between blocks for either practice group. Subsequently, we performed a simple effects analysis to analyze the effect of group at each level of the block factor. This analysis yielded significant results for blocks 13 (*p =* 0.033), 15 (*p* = 0.008), 17 (*p* = 0.039), 18 (*p* = 0.024), 19 (*p* = 0.019), and 20 (*p* = 0.016). In all cases, the number of samples outside the bandwidth was greater for the error augmentation group. This suggests that in the last quarter of acquisition, the error augmentation group received significantly more haptic feedback than the error minimization group.

### Performance during Acquisition with Various Forms of Haptic Feedback

**FIGURE 3:**
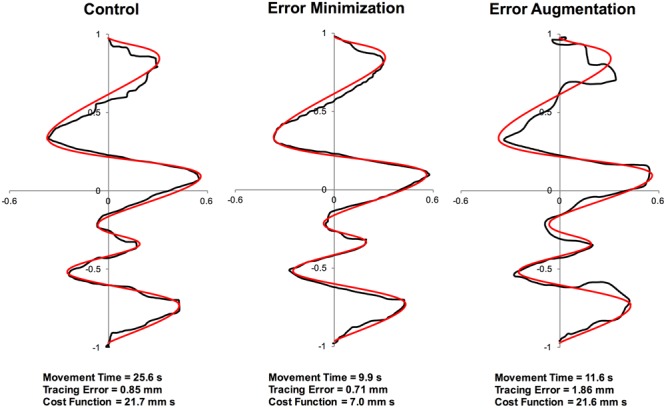
**Traces of the fifth acquisition trial from a representative participant in each practice group (black curves) alongside the target curve shown in red**.

**FIGURE 4:**
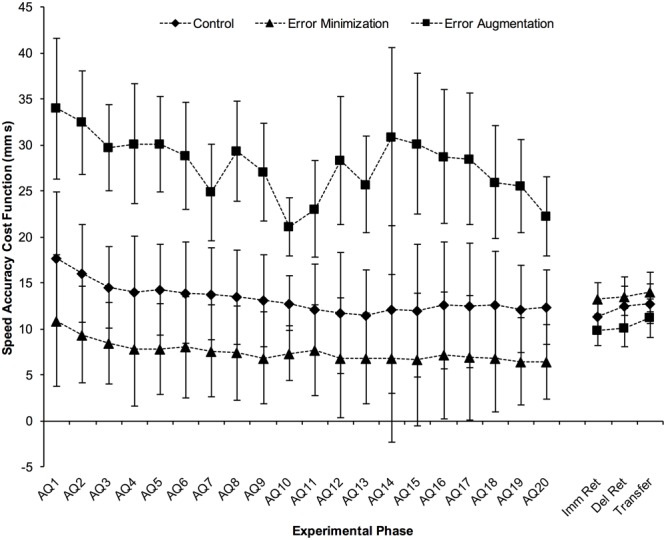
**Mean Speed Accuracy Cost Function by group and experimental phase (block in acquisition and test in retention/transfer)**. Error bars are 95% confidence intervals. AQ = acquisition, Imm Ret = immediate retention, Del Ret = delayed retention.

**FIGURE 5:**
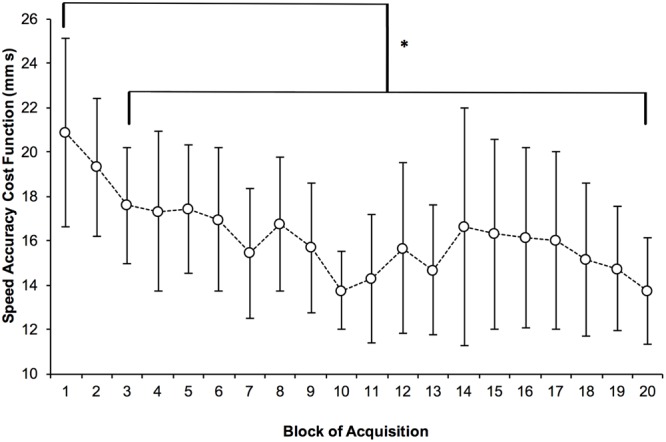
**Grand Means of Speed Accuracy Cost Function by block of acquisition.** Error bars are 95% confidence intervals, ^∗^*p* < 0.05.

Representative traces from a participant in each group are shown in **Figure 3**, along with the corresponding tracing error, movement time and cost function for those trials. **Figure 4** summarizes the results of speed accuracy cost function for each group during skill acquisition and tests and learning. Mauchly’s test of sphericity was significant, χ^2^(189) = 625.6, *p* < 0.001, so degrees of freedom were corrected using Greenhouse-Geisser estimates. There was a main effect of block, *F*(4.2,96.7) = 7.0, *p* < 0.001, η_p_^2^ = 0.23 (**Figure 5**) and contrasts revealed that cost function on acquisition block 1 was significantly higher than cost function on block 3 and all subsequent blocks of acquisition (all *p* < 0.05).

**FIGURE 6:**
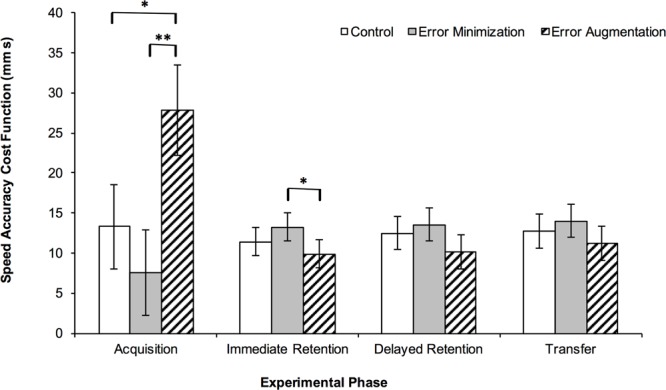
**Mean Speed Accuracy Cost Function by practice group in each experimental phase.** Error bars are 95% confidence intervals, ^∗^*p* < 0.05 and ^∗∗^*p* < 0.001.

There was also a main effect of practice group, *F*(2,23) = 15.4, *p* < 0.001, η_p_^2^ = 0.57 (**Figure 6**), and Tukey HSD *post hoc* comparisons showed that the error augmentation group performed worse (i.e., higher cost function) during acquisition than both the error minimization group (*p* < 0.001) and the control group (*p* = 0.002). As might be expected, the error augmentation group also exhibited greater variance than the control and error minimization groups. Consequently, we sought to confirm our *post hoc* comparisons by using Games-Howell *post hoc* comparisons. These comparisons confirmed the group differences described above but also suggested that the control and error minimization groups were significantly different (*p* < 0.001).

#### Contributions of Movement Time and Tracing Error to Acquisition Performance

To better understand the main effect of practice group for overall performance as indicated by the speed accuracy cost function, we conducted separate mixed ANOVAs on movement time and tracing error. Similar to the cost function analysis, there were main effects of practice group for both movement time, *F*(2,23) = 7.8, *p* = 0.003, η_p_^2^ = 0.41, and tracing error data, *F*(2,23) = 11.1, *p* < 0.001, η_p_^2^ = 0.49 (see **Table 1**). Tukey HSD *post hoc* comparisons indicated that for movement time, the error minimization group had significantly faster movement time than both control (*p* = 0.003) and error augmentation (*p* = 0.016) groups, while for tracing error, the error augmentation group had significantly worse tracing error than the control and error minimization groups (both *p* = 0.001). However, the main effect of block observed for cost function was reflective only of participants’ movement time: there was a main effect of block for movement time, *F*(3.3,74.9) = 17.3, *p* < 0.001, η_p_^2^ = 0.43, but none for tracing error, *F*(3.1,71.4) = 1.6, *p* = 0.196, η_p_^2^ = 0.07 (see **Table 2**). This suggests that participants improved their performance over the course of acquisition by maintaining their tracing error but improving their movement time (i.e., tracing faster).

**Table 1:** Mean values of movement time and tracing error for each practice group during skill acquisition.

Practice group	Movement time (s)		Tracing error (mm)	
	Mean	95% CI	Mean	95% CI
Control	16.4	[13.7, 19,1]	0.85	[0.44, 1.26]
Error minimization	9.6	[6.9, 12.3]	0.82	[0.41, 1.23]
Error augmentation	15.3	[12.5, 18.2]	2.03	[1.59, 2.46]

**Table 2:** Mean values of movement time and tracing error for each block of skill acquisition.

Block of acquisition	Movement time (s)		Tracing error (mm)	
	Mean	95% CI	Mean	95% CI
1	18.3	[16.0, 20.7]	1.23	[0.94, 1.51]
2	17.8	[15.3, 20.3]	1.18	[0.94, 1.42]
3	16.2	[13.8, 18.6]	1.18	[0.92, 1.43]
4	16.1	[13.9, 18.3]	1.12	[0.86, 1.39]
5	14.8	[12.9, 16.6]	1.20	[1.00, 1.40]
6	14.7	[12.9, 16.5]	1.17	[0.96, 1.38]
7	14.4	[12.6, 16.1]	1.18	[0.87, 1.49]
8	14.4	[12.3, 16.5]	1.18	[0.98, 1.38]
9	12.8	[11.4, 14.2]	1.31	[0.99, 1.64]
10	13.0	[11.2, 14.8]	1.10	[0.92, 1.29]
11	13.2	[11.3, 15.1]	1.14	[0.90, 1.38]
12	12.7	[10.9, 14.4]	1.29	[0.94, 1.65]
13	12.3	[10.7, 14.0]	1.30	[0.95, 1.64]
14	12.4	[10.8, 13.9]	1.32	[0.98, 1.66]
15	12.4	[10.8, 14.0]	1.30	[1.07, 1.53]
16	12.5	[10.9, 14.0]	1.29	[1.01, 1.56]
17	12.3	[10.5, 14.1]	1.37	[1.02, 1.72]
18	11.8	[10.2, 13.4]	1.33	[1.06, 1.59]
19	11.6	[10.0, 13.1]	1.31	[1.05, 1.57]
20	12.0	[10.4, 13.6]	1.20	[0.99, 1.41]

### Performance on Retention and Transfer Tests

**Figure 6** shows group means of speed accuracy cost function for each of the retention and transfer tests. Analysis of cost function for the retention and transfer tests showed that there was a main effect of group for the immediate retention test, *F*(2,24) = 4.0, *p* = 0.031, η_p_^2^ = 0.25, and Tukey HSD *post hoc* comparisons indicated that the error minimization group performed significantly worse than the error augmentation group, *p* = 0.024. There were no statistically significant effects of group for either the delayed retention test, *F*(2,24) = 2.9, *p* = 0.076, η_p_^2^ = 0.19, or the transfer test, *F*(2,24) = 1.9, *p* = 0.173, η_p_^2^ = 0.14.

#### Contributions of Movement Time and Tracing Error to Overall Performance on Retention and Transfer Tests

**FIGURE 7:**
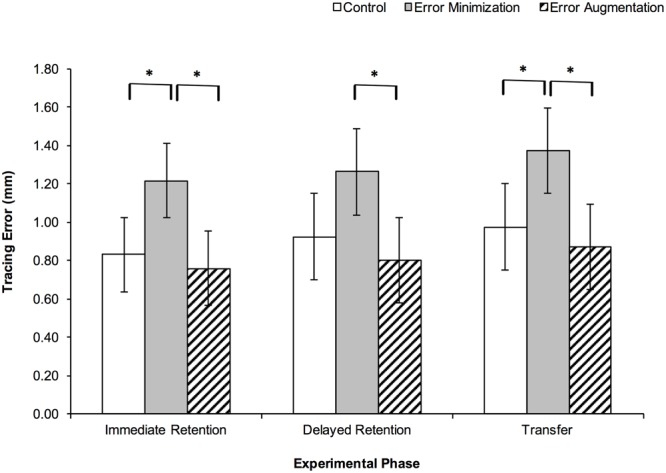
**Mean Tracing Error by practice group for each retention and transfer test.** Error bars are 95% confidence intervals, ^∗^*p* < 0.05.

Further analysis of movement time and tracing error indicated that the observed effects on cost function were due primarily to accuracy rather than speed. Movement time data yielded no main effects of group for any of the tests: immediate retention, *F*(2,24) = 0.6, *p* = 0.574, η_p_^2^ = 0.05; delayed retention, *F*(2,24) = 0.3, *p* = 0.743, η_p_^2^ = 0.02; transfer, *F*(2,24) = 0.5, *p* = 0.599, η_p_^2^ = 0.04. The mean movement times across all groups, were 13.6 s (*SD* = 4.6), 13.3 s (*SD* = 4.9), and 12.9 s (*SD* = 4.3) for the immediate retention, delayed retention and transfer tests, respectively. In contrast, analysis of the tracing error data revealed main effects of group for all retention and transfer tests: immediate retention, *F*(2,24) = 6.8, *p* = 0.005, η_p_^2^ = 0.36; delayed retention, *F*(2, 24) = 4.9, *p* = 0.017, η_p_^2^ = 0.29; and transfer, *F*(2,24) = 5.9, *p* = 0.008, η_p_^2^ = 0.33 (**Figure 7**). *Post hoc* Tukey HSD comparisons indicated that for the immediate retention test, the error minimization group was less accurate than both the control (*p* = 0.021) and error augmentation (*p* = 0.006) groups; for the delayed retention test, the error minimization group was less accurate than the error augmentation group (*p* = 0.016); and for the transfer test, the error minimization group was less accurate than both the control (*p* = 0.042) and error augmentation (*p* = 0.009) groups. Because there was some concern regarding equality of group variances for the transfer test, we also used Games-Howell *post hoc* comparisons, which called into question the significance of the difference between the error minimization and control groups (*p* = 0.094).

## Discussion

We asked participants to practice a curve-tracing task while being subjected to one of three conditions of haptic feedback regarding their tracing error: no augmented haptic feedback (control), error minimization or error augmentation. During skill acquisition and on retention and transfer tests, we instructed participants to trace the curve as quickly and as accurately as possible. We tested all participants, in the absence of any augmented feedback, at two times points – immediately after and 1 day after the acquisition phase. We measured movement time, tracing error, and a composite performance variable – speed accuracy cost function – to determine which practice condition best facilitated learning of the task. While the immediate retention test showed immediate but transient effects of the practice conditions on post-training performance, learning was specifically inferred from performance on the delayed retention and transfer tests. For the speed accuracy cost function, we only observed group differences immediately after practice: the error minimization group showed worse performance than the error augmentation group. While there were no group differences in movement time on any of the tests, there were significant group differences in tracing error for all retention and transfer tests. We observed that the error minimization group was less accurate than both the control and error augmentation groups on both the immediate retention and transfer tests but less accurate than only the error augmentation group on the delayed retention test. It is important to note that the difference between the error minimization and control groups did not persist the day after training. This highlights the fact that conclusions drawn from tests immediately after practice are insufficient to infer learning. Overall, our results indicate that the error augmentation group learned more than the error minimization group. These results provide partial support for our hypotheses.

### Type of Task, Training Parameters, and Learning

One primary reason for the inconclusive results regarding the benefits of haptic training is that researchers have not clearly differentiated or discussed the features of the various motor tasks or training programs that might impact their results (Powell and O’Malley, 2012; Heuer and Lüttgen, 2015). In addition to the basic difference between error minimizing and error augmenting types of haptic feedback, we have previously identified a subtle but important difference between implementations of haptic assistance, namely assistance as demonstration versus assistance as performance feedback (Williams and Carnahan, 2014), which likely invoke different mechanisms of motor learning (Heuer and Lüttgen, 2015). Researchers in (non-robotic) motor learning have long recognized the importance of these relationships among type of tasks, feedback parameters/practice conditions and motor learning. Singer (1977) noted that self-paced, closed skills that are performed in a stable environment, are generally targeted to automation. As such, reducing errors during practice, which encourages repetition of the correct performance, with minimal use of feedback, should be most beneficial for learning such tasks. In contrast, externally paced, open skills should be practiced for adaptability to a dynamic environment and should benefit more from the varied experience of errors during practice (Singer, 1977). However, our results do not provide support for this assertion, as learning of our self-paced, closed skill did not benefit from error minimization during practice. One explanation could be that the task was not *strictly* self-paced because participants were instructed to move as quickly and as accurately as possible (Kreifeldt, 1972). Outside the laboratory, very few skills are strictly self-paced; that is, most functional self-paced skills (e.g., driving) do have some implicit or explicit guidelines for movement speed. Thus, the instructions utilized in the present study may be more ecologically valid for real-world, closed skills.

In a more recent review, Heuer and Lüttgen (2015) take a neuro-cognitive perspective to address these relationships, specifically in the context of robot assistance for motor learning. Using the presented classification scheme for the products of motor learning, our task can be described as requiring spatial trajectory learning (Heuer and Lüttgen, 2015). While other tracking or path-following tasks, such as drawing circles (Viviani and Terzuolo, 1982) and steering vehicles (Marchal-Crespo and Reinkensmeyer, 2008), may also include dynamic features of trajectory learning, we did not require participants to adhere to any particular velocity or timing-based profile. We measured movement time in order to observe and account for the speed-accuracy trade-off inherent in this type of self-paced task. Importantly, Heuer and Lüttgen (2015) indicate that when a trajectory is demonstrated (visually or haptically), the primary mechanism of motor learning is probably observational learning, while error-based or reward-based learning mechanisms are invoked when error feedback is available, as it was in our experiment. This is along the same lines of the distinction we drew between haptic demonstration and haptic feedback (Williams and Carnahan, 2014). Based on the current literature, they go on to suggest that when error-based learning is the primary mechanism of motor learning, convergent (i.e., error minimizing) haptic training should inhibit learning while divergent (i.e., error augmenting) haptic training should facilitate learning. They also deduced that, based on current evidence, error augmentation may work best for tasks that involve simple paths and require precise hand movements. Our present results – the benefit of error augmentation for learning a simple path requiring precise hand movements – support both these conclusions.

### The Benefit of Increased Errors during Skill Acquisition

Our data clearly indicate that the error augmentation group experienced significantly more errors during skill acquisition than both the error minimization and control groups. Consistent with the predictions made by Heuer and Lüttgen (2015) as well as error-driven models of motor learning, the error augmentation group exhibited the best performance on tests of learning. We propose that this group’s extensive experience with error-correction during skill acquisition strengthened the internal model for the task and led to greater learning (Thoroughman and Shadmehr, 2000; Wolpert and Ghahramani, 2000). Our results are also consistent with previous studies of physical guidance, which reported that practice conditions that provide frequent on-target experience (such as our error minimization condition) were detrimental for learning (Winstein et al., 1994; Sidaway et al., 2008). In the study by Winstein et al. (1994), participants who practiced an angular positioning task with a physical block reported that they moved quickly to the target without concentrating on stopping at the target location. The study authors proposed that this approach to practice disrupted information processing activities that are crucial for skill retention (Winstein et al., 1994). Similarly, it is possible that our error minimization participants simply traced the curve quickly without focusing much on accuracy because they knew that the device would keep them on target. This interpretation is compatible with theories suggesting that more effortful training conditions lead to better learning (Bjork, 1994; Linn and Bjork, 2006) as well as the notion of deliberate practice required to attain expertise in a given skill (Ericsson et al., 1993). It is also interesting to note that the control group exhibited longer movement times than the error minimization group during skill acquisition. This suggests that, despite their errors being similar in magnitude to that of the error minimization group during skill acquisition, the control group may have exhibited more effort during practice. Indeed, the longer movement times can be attributed to increased effort devoted to ensuring that there were very few errors in their movement. In other words, although the control group did not experience large errors, they were actively engaged in error detection and error correction that were reflected in longer movement times. This effortful but largely errorless approach to skill acquisition may have led to the observed trends for the control group’s better accuracy in comparison to the error minimization group on retention and transfer tests. However, since we did not measure attention, motivation or effort, we cannot confirm these suppositions.

Another potential reason for the observed benefits of error augmentation is that divergent force fields and random perturbations are known to cause limb stiffness which can result in more precise movements due to co-contraction of muscles (Heuer and Lüttgen, 2015). This is also supported by the observation that random perturbations (and not just feedback related error augmenting forces) can facilitate performance improvement (Marchal-Crespo et al., 2014a) and motor learning of spatial movement characteristics (Lee and Choi, 2010).

Comparing our results with other studies of haptic training is a little difficult given the differences between our experiment and others that targeted spatial features of trajectories and included evaluation of a haptic error augmenting training condition. Tasks in such studies included pursuit tracking around the outline of a figure (Lee and Choi, 2010; Powell and O’Malley, 2012), point-to-point movements (Burdet et al., 2001; Su et al., 2011), as well as steering a simulated vehicle (Lee and Choi, 2014) and a wheelchair with a joystick (Chen and Agrawal, 2013). Results for the studies utilizing pursuit tracking are mixed: Powell and O’Malley (2012) observed no group differences for performance immediately after practice while Lee and Choi (2010) observed that noise-like haptic disturbance as well as repulsive (feedback-based) haptic disturbance both resulted in better learning (as observed on a delayed retention test) when compared to progressive haptic guidance. The primary difference between these tasks and ours is that our participants did not have to follow a fixed target speed, they were free to trade-off speed for spatial accuracy. Both point-to-point movement tasks showed benefits of practicing in error augmenting force fields for producing straight paths after the force fields were removed (Burdet et al., 2001; Su et al., 2011). However, it could be argued that this ballistic task does not provide the same online visual feedback information that characterizes our task and other path-following tasks. Results of the steering tasks were not entirely consistent with each other: Lee and Choi (2014) reported no differences in tracking errors between their control, progressive guidance, haptic disturbance and hybrid guidance/disturbance groups on a delayed retention test while Chen and Agrawal (2013) reported that their assistive and resistive force groups both outperformed their control group, but did not differ from each other, immediately after training. One likely reason for this inconsistency is the difference in dynamics between the two steering tasks. Nonetheless, it is interesting to note that Lee and Choi’s (2014) simulated vehicle was steered at a constant speed while Chen and Agrawal’s (2013) participants were instructed to travel at the maximum speed but, ultimately, could control the wheelchair’s speed. Even though both steering tasks were evaluated only on spatial accuracy, both sets of authors acknowledge that in addition to the spatial, error-cancelling aspect of performance, a dynamic feature of the task, specifically the timely initiation of turns, was essential to success. Similar dynamic features are likely involved in our task but we did not explicitly train or measure these features. Overall, our contribution to this body of literature is evidence that error augmentation provides benefits over error minimization for learning spatial accuracy, even when participants can trade speed for accuracy.

### The Detriment of Increased Error Feedback during Skill Acquisition

It is known that feedback can provide motivation and information to guide future performance and, as such, more feedback can mean more information to enhance learning. However, studies have shown that learners can come to depend on increased amounts of feedback and ignore processing of other information (e.g., intrinsic feedback) that would contribute to learning and the development of error-detection capabilities (Salmoni et al., 1984; Schmidt, 1991). These findings are summarized by the guidance hypothesis which predicts that too much guidance will lead to enhanced performance during skill acquisition but worse performance during retention tests. The error augmenting condition, could not, by definition and design, lead to improved performance during skill acquisition (i.e., provide guidance). We will therefore consider only the control and error minimization groups when discussing the relationship between learning and the amount of feedback received in skill acquisition.

The amount of feedback experienced by participants in the error minimization group can be quantified via the proportion of samples/trial for which a participant received haptic feedback, i.e., the proportion of samples/trial that occurred outside the haptic feedback bandwidth. The amount of feedback that this group received was more or less constant throughout practice, averaging 45% of each trial in the first quarter of acquisition trials and 43% in the last quarter. Naturally, the amount of haptic feedback experienced by the control group was nil throughout skill acquisition. Other studies utilizing physical guidance have noted that the augmented information provided by physical guidance tends to be more guiding than feedback in the form of knowledge of results (as the control group received) because physical guidance has typically worked directly toward error reduction and successful task performance (Winstein et al., 1994). However, our results are inconclusive with respect to providing support for the guidance hypothesis: the control group was only significantly better than the error minimization on the transfer test, and only for tracing error, not speed accuracy cost function. Superior performance on transfer indicates that the skills acquired during training have generalized to benefit performance on variations of the task. However, there was no difference between these groups on the delayed retention test and the Games-Howell *post hoc* procedure indicated that these groups were not different on the transfer test.

Another potential explanation for the error minimization group’s learning effects is that their practice formed an internal model of the task that *included* the dynamics of error minimization (Armstrong, 1970; Schmidt, 1991). As such, their internal model for the basic, unguided task was undeveloped and when they were asked to perform the task without any augmented feedback, minimal learning was observed. In contrast, the control group was tested under the same conditions as practice and so their internal model of the task was perfectly suited to the testing conditions and it would have been difficult for the error augmentation group to incorporate the dynamics of haptic feedback into their internal model of the task because the feedback they experienced was so disruptive.

### The Utility of Error-Altering Haptic Feedback

We have demonstrated that there is benefit to using error augmenting haptic feedback over error minimizing haptic feedback for tracing a two-dimensional curve. Ultimately, however, our results indicated that the control (no haptic feedback) group’s learning was not significantly different from either of the experimental groups. Similar results have been observed in other studies (Lee and Choi, 2010; Marchal-Crespo et al., 2014b). This could indicate that error-altering haptic feedback is unnecessary for this task, perhaps due to its simplicity. However, it is possible that what error-altering feedback, and more specifically error augmenting feedback offers is greater efficiency in learning a task. Most studies of haptic training are designed with a view to future applications to fields such a robotic rehabilitation or surgical training where saving time and money are key factors when introducing new training programs. As such, researchers should not only seek to determine which error-altering haptic feedback strategies facilitate learning but also whether there are differences in the time required to achieve *and maintain* the desired level of performance.

## Conclusion

Our study has contributed to the emerging body of literature that is exploring opposing forms of error-altering haptic feedback for learning a self-paced, trajectory-based skill. Our results showed that participants who experienced haptic error augmentation during skill acquisition learned more than those who experienced haptic error minimization. In particular, we have shown that haptic error augmentation is more beneficial than error minimization for learning spatial accuracy, even when learners have full control of the speed of performance. The error minimization group, which received more feedback than the control group during skill acquisition, tended to perform worse than the control group on retention tests but this difference was not significant on the delayed retention test (cf. Guidance hypothesis: Salmoni et al., 1984; Schmidt, 1991). While the overall performance of the control group was not different from the error augmentation group on any tests, the error augmentation group was consistently more accurate than the error minimization group on retention as well as transfer tests. We believe that this was due to the nature of the error augmenting haptic feedback, which impeded successful task performance and provided a rich experience of error detection and correction processes during skill acquisition. Additionally, although the control group did not experience large errors during skill acquisition, this group also likely engaged in effortful practice (as inferred from the relationship between their movement time and tracing errors), which allowed them to achieve levels of accuracy comparable to the error augmentation group on tests of learning. Taken together, our results suggest that the effortful detection and self-initiated correction of errors during practice can be more important than accurately-guided practice for the learning of curve-tracing. These findings should be extended to studies testing the design and long-term learning implications of physical guidance protocols in various practical settings, such as physical rehabilitation and sports.

## Ethics Statement

This study was approved by Health Sciences Research Ethics Board, University of Toronto. Prospective participants were provided with an electronic copy of the information letter and consent form as well as further details regarding inclusion and exclusion criteria, time requirement and how to schedule participation. The information letter outlined the general purpose of the research, the research procedure, as well as risks and anticipated benefits. In addition, it contained a statement offering participants the opportunity to ask questions and to withdraw at any time from the research procedures as well as contact information for the study investigators and the Director of the Office of Research Ethics who was not involved or associated with the study. Once a prospective participant arrived at the lab with the intention to participate, the experimenter explained the study procedures then allowed the participant to read a paper copy of the information letter and consent form and to ask any questions. If they agreed to participate, they were then asked to sign the consent form. The experimenter also signed, then collected, the completed consent form.

## Author Contributions

CW and HC were responsible for the conception and design of the work. CW was primarily responsible for data acquisition, analysis, and interpretation with support from LT and HC for data analysis and interpretation. CW drafted the manuscript while HC and LT provided critical revisions and approved the final version of the manuscript.

## Conflict of Interest Statement

The authors declare that the research was conducted in the absence of any commercial or financial relationships that could be construed as a potential conflict of interest.

## Footnotes

1 http://www.randomizer.org/
